# Prescription of Anticholinergic Drugs in Patients With Schizophrenia: Analysis of Antipsychotic Prescription Patterns and Hospital Characteristics

**DOI:** 10.3389/fpsyt.2022.823826

**Published:** 2022-05-17

**Authors:** Hikaru Hori, Norio Yasui-Furukori, Naomi Hasegawa, Jun-ichi Iga, Shinichiro Ochi, Kayo Ichihashi, Ryuji Furihata, Yoshitaka Kyo, Yoshikazu Takaesu, Takashi Tsuboi, Fumitoshi Kodaka, Toshiaki Onitsuka, Tsuyoshi Okada, Atsunobu Murata, Hiroko Kashiwagi, Hitoshi Iida, Naoki Hashimoto, Kazutaka Ohi, Hisashi Yamada, Kazuyoshi Ogasawara, Yuka Yasuda, Hiroyuki Muraoka, Masahide Usami, Shusuke Numata, Masahiro Takeshima, Hirotaka Yamagata, Tatsuya Nagasawa, Hiromi Tagata, Manabu Makinodan, Mikio Kido, Eiichi Katsumoto, Hiroshi Komatsu, Junya Matsumoto, Chika Kubota, Kenichiro Miura, Akitoyo Hishimoto, Koichiro Watanabe, Ken Inada, Hiroaki Kawasaki, Ryota Hashimoto

**Affiliations:** ^1^Department of Psychiatry, Faculty of Medicine, Fukuoka University, Fukuoka, Japan; ^2^Department of Psychiatry, Dokkyo Medical University School of Medicine, Tochigi, Japan; ^3^National Institute of Mental Health, National Center of Neurology and Psychiatry, Kodaira, Japan; ^4^Department of Neuropsychiatry Molecules and Function, Ehime University Graduate School of Medicine, Matsuyama, Japan; ^5^Department of Neuropsychiatry, University of Tokyo Hospital, Tokyo, Japan; ^6^Agency for Student Support and Disability Resources, Kyoto University, Kyoto, Japan; ^7^Department of Psychiatry, School of Medicine, Kitasato University, Tokyo, Japan; ^8^Department of Neuropsychiatry, Graduate School of Medicine, University of the Ryukyus, Nishihara, Japan; ^9^Department of Neuropsychiatry, Kyorin University School of Medicine, Mitaka, Japan; ^10^Department of Psychiatry, The Jikei University School of Medicine, Tokyo, Japan; ^11^Department of Neuroimaging Psychiatry, Graduate School of Medical Sciences, Kyushu University, Fukuoka, Japan; ^12^Department of Psychiatry, Jichi Medical University, Tochigi, Japan; ^13^Department of Psychiatry, National Center of Neurology and Psychiatry Hospital, Kodaira, Japan; ^14^Department of Psychiatry, Hokkaido University Graduate School of Medicine, Sapporo, Japan; ^15^Department of Psychiatry, Gifu University Graduate School of Medicine, Gifu, Japan; ^16^Department of Neuropsychiatry, Hyogo College of Medicine, Nishinomiya, Japan; ^17^Center for Postgraduate Clinical Training and Career Development, Nagoya University Hospital, Nagoya, Japan; ^18^Life Grow Brilliant Mental Clinic, Medical Corporation Foster, Osaka, Japan; ^19^Department of Psychiatry, Tokyo Women’s Medical University, Tokyo, Japan; ^20^Department of Child and Adolescent Psychiatry, Kohnodai Hospital, National Center for Global Health and Medicine, Ichikawa, Japan; ^21^Department of Psychiatry, Graduate School of Biomedical Science, Tokushima University, Tokushima, Japan; ^22^Department of Neuropsychiatry, Akita University Graduate School of Medicine, Akita, Japan; ^23^Division of Neuropsychiatry, Department of Neuroscience, Yamaguchi University School of Medicine, Yamaguchi, Japan; ^24^Department of Neuropsychiatry, Kanazawa Medical University, Uchinada, Japan; ^25^Department of Neuropsychiatry, Toho University Graduate School of Medicine, Tokyo, Japan; ^26^Department of Psychiatry, Nara Medical University School of Medicine, Kashihara, Japan; ^27^Toyama City Hospital, Toyama, Japan; ^28^Department of Neuropsychiatry, University of Toyama Graduate School of Medicine and Pharmaceutical Sciences, Toyama, Japan; ^29^Katsumoto Mental Clinic, Oosaka, Japan; ^30^Department of Psychiatry, Tohoku University Hospital, Sendai, Japan; ^31^Department of Psychiatry, Yokohama City University Graduate School of Medicine, Yokohama, Japan

**Keywords:** schizophrenia, anticholinergic drugs, antipsychotic polypharmacy, antipsychotic monotherapy, first-generation antipsychotics, second-generation antipsychotic (SGA)

## Abstract

In several clinical guidelines for schizophrenia, long-term use of anticholinergic drugs is not recommended. We investigated the characteristics of the use of anticholinergics in patients with schizophrenia by considering psychotropic prescription patterns and differences among hospitals. A cross-sectional, retrospective prescription survey at the time of discharge was conducted on 2027 patients with schizophrenia from 69 Japanese hospitals. We examined the relations among psychotropic drug prescriptions regarding anticholinergic prescription. We divided the hospitals into three groups—low rate group (LG), medium rate group (MG), and high rate group (HG)—according to their anticholinergic prescription rates, and analyzed the relationship between anticholinergic prescription rates and antipsychotic prescription. Anticholinergic drugs were prescribed to 618 patients (30.5%), and the prescription rates were significantly higher for high antipsychotic doses, antipsychotic polypharmacy, and first-generation antipsychotics (FGAs) use. The anticholinergic prescription rate varied considerably among hospitals, ranging from 0 to 66.7%, and it was significantly higher in patients with antipsychotic monotherapy, antipsychotic polypharmacy, and normal and high doses of antipsychotics in HG than in those LG and MG. The anticholinergics prescription rate in patients with second-generation antipsychotic monotherapy in HG was also significantly higher than in those LG and MG; however, the difference was no longer significant in patients with FGA monotherapy. Conclusively, in addition to high antipsychotic doses, antipsychotic polypharmacy, and FGA use, hospital characteristics influence the prescribing of anticholinergic drugs.

## Introduction

Schizophrenia is one of the top 15 causes of health burden in the world (1). The characteristics of its clinical presentation are positive and negative symptoms and cognitive impairment (2). In recent years, the goal of treatment for schizophrenia has been recovery, which requires improvement in the level of functioning, including cognitive dysfunction (3–8). Antipsychotic medication is the mainstay for managing schizophrenia, and psychosocial treatments, including cognitive-behavioral therapy, social skills training, assertive community treatment, supported employment, occupational therapy (9), and teaching illness, are helpful as well.

Antipsychotics induce a blockade of the dopamine D2 receptors, and this antagonism in the limbic system may be responsible for the effect on positive symptoms, whereas the antagonism of dopamine transmission in the basal ganglia causes extrapyramidal symptoms (EPS). In clinical practice, anticholinergics are widely used for treating and preventing EPS induced by antipsychotic use (10). In general, first-generation antipsychotics (FGAs) are prone to cause EPS, whereas this risk decreases in the case of second-generation antipsychotics (SGAs), whereas antipsychotic polypharmacy causes a higher EPS rate than antipsychotic monotherapy (11). Moreover, studies have shown that antipsychotic-induced EPS is mostly dose-dependent (12).

Anticholinergics are also known to be associated with peripheral side effects, such as urinary disturbances, dry mouth, and constipation. Moreover, its main adverse effects are often associated with impaired cognitive functioning in schizophrenia (13–15). Therefore, in current treatment guidelines for schizophrenia, prophylactic use of anticholinergics is not recommended in the long term (13, 16–18). The high use rate of long-term concurrent anticholinergics with antipsychotics has been noted as an important therapeutic issue in some countries. For instance, Xiang et al. (19) found that anticholinergics were administered to more than half of the considered schizophrenia patients in 2009. In addition, a previous Japanese study reported that adjunctive anticholinergics were prescribed to 56–75% of chronic schizophrenia inpatients even though they were taking SGAs (20). A European study performed in six countries found that 30% of patients treated with SGAs were also prescribed anticholinergics (21). Based on these findings, one can conclude that significant discrepancies between the recommended treatment guidelines and real-world clinical practice in schizophrenia exist globally (22, 23).

To disseminate and implement uniform clinical guidelines, the Effectiveness of Guidelines for Dissemination and Education in Psychiatric Treatment (EGUIDE) project was started in 2016 (24–31). The EGUIDE project started with the cooperation of 22 hospitals, and as of 2021, more than 240 hospitals (44 universities) have participated. In brief, the EGUIDE project disseminates the guidelines through lectures and case discussions. Previous reports from the EGUIDE project have shown that there are large interinstitutional differences in prescribing treatment patterns for schizophrenia and major depressive disorders (24, 25, 26). We hypothesized that this would be the case for anticholinergic prescriptions as well, with large variations in prescribing patterns from hospital to hospital, influenced by the underlying antipsychotic prescriptions.

In this study, we analyzed the anticholinergic prescriptions for patients with schizophrenia in Japan using data at the time of discharge, further classified the patients into three groups according to the anticholinergic prescription rates at each hospital, and then compared them in terms of antipsychotic dosage, antipsychotic polypharmacy/monotherapy, and FGA/SGA monotherapy.

## Materials and Methods

### Study Design

Using data from the EGUIDE project, a cross-sectional pharmacological survey was first conducted in 2016. A survey was conducted on all psychotropics on patients with schizophrenia who were discharged from participating hospitals between April and September before the educational program (25, 26, 32).

### Participants

**FIGURE 1:**
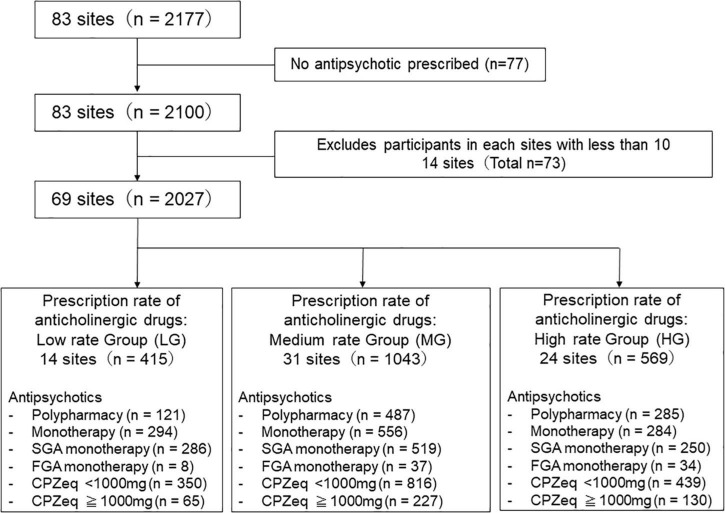
Study flow chart. The institutions were divided into three groups according to the anticholinergics prescription rate.

In total, 83 hospitals participated in this survey. A cross-sectional case record survey was conducted using standardized data collection from each study site, involving a sample of 2,177 patients who had been diagnosed with schizophrenia according to the Diagnostic and Statistical Manual of Mental Disorders, 5th Edition (DSM-5) criteria at the time of discharge. As our study aimed to investigate anticholinergics prescription status, 77 patients who were not administered antipsychotics were excluded from the analysis. Hospitals with <10 discharges during the study period were also excluded (14 hospitals, 73 patients). Finally, a total of 69 hospitals (33 university hospitals, 19 national/public hospitals, and 17 private hospitals) were included in this study (Figure 1). In this work, we analyzed data from the first year before joining the EGUIDE program from 2016 to 2018.

### Methods

The EGUIDE project members collected the data. Age, gender, and clinical characteristics of the patients were collected. Information about the types and doses of all antipsychotic drugs was collected from medical records. Doses of antipsychotic drugs were converted into approximate chlorpromazine equivalent milligrams (CPZeq) (33). Based on previous reports (34), we defined the high-dose and normal-dose groups as ≥1,000 mg/day and <1,000 mg/day of CPZeq for antipsychotics, respectively. We also used biperiden equivalent (BPeq). Anticholinergics include amantadine, benztropine, biperiden, mazaticol, procyclidine, promethazine, and trihexyphenidyl.

The hospitals were divided into three groups according to the anticholinergic prescription rate. In the EGUIDE project, we aimed to reduce the prescription rate of anticholinergic drugs to <20%. To see the educational effect, we categorized the prescribing groups by hospital prescribing rate of 20%. We defined and categorized into three groups [low rate group (LG) (0≤% of anticholinergic prescription <20), medium rate group (MG) (20=% of anticholinergic prescription <40), and high rate group (HG) (40<%anticholinergic prescription)]. We compared the use of antipsychotics dosage, antipsychotics monotherapy/polypharmacy, and FGA/SGA monotherapy among these three groups of patients according to the anticholinergics prescription rate.

The ethics committees of the National Center of Neurology and Psychiatry and each participating university/hospital/clinic approved the entire study protocol, and the study was carried out in accordance with the latest version of the Declaration of Helsinki. Patients were able to opt-out of the purpose and procedures of the study and refuse study participation. The study protocol has been registered in the University Hospital Medical Information Network Registry (UMIN000022645).

### Statistical Analysis

All statistical analyses were performed using SPSS software for Windows (Version 26.0). The data are presented as mean ± standard deviations. We used the Shapiro–Wilk test to assess the data correspondence to the normal distribution. Demographic characteristics and the results were compared among the groups. The anticholinergic prescription rate was calculated as: schizophrenic patients prescribed anticholinergics at discharge/all discharged schizophrenia patients. Spearman’s correlation analysis was used to determine the correlation of the dosages of antipsychotics (CPZeq) and anticholinergics (BP). The descriptive statistics were tabulated using either the Chi square test or analysis of variance to test for significant differences across the groups with *Post-hoc* Bonferroni. The level of statistical significance was set at *p* < 0.05. For multiple comparison test, a *p*-value of <0.033 (0.05/3) was considered significant.

## Results

We enrolled 2,027 patients diagnosed with schizophrenia (924 males and 1,103 females). All baseline demographic data are summarized in Table 1. In this survey, 618 participants (30.5%) were administered with anticholinergics, the most common being biperiden (*n* = 493), trihexyphenidyl (*n* = 86), and promethazine (*n* = 68). A comparison of anticholinergic prescription rates for the Top 3 of these groups, biperiden, trihexyphenidyl, and promethazine, is shown in Supplementary Table 1. Prescribing rates for all three anticholinergics were higher for high antipsychotic doses, antipsychotic polypharmacy, and FGA use.

**TABLE 1:** Demographics data of the study.

Age (year) *M* (SD)	45.7 (15.2)
Gender (M/F)	924/1103
CPZeq (mg/day) *M* (SD)	683.5 (449.1)
Antipsychotic polypharmacy rate (%)	44.1
BPeq (mg/day) *M* (SD)	0.8 (1.5)
Anticholinergics prescription rate (%)	30.5
Antidepression prescription rate (%)	8.5
Mood stabilizers or anticonvulsants prescription rate (%)	35.6

*CPZeq, chlorpromazine equivalent; BPeq, biperiden equivalent; M, mean, SD, standard deviation.*

**FIGURE 2:**
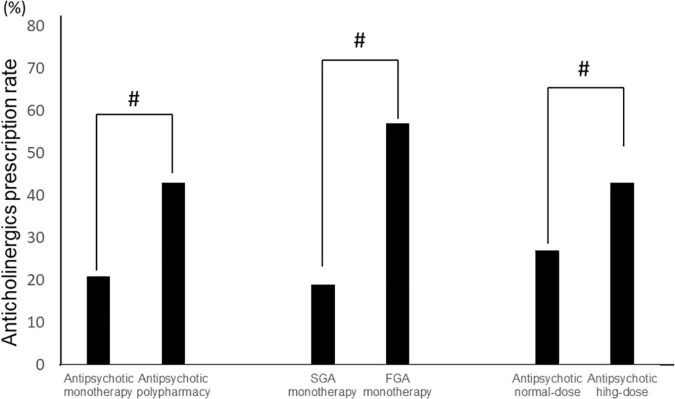
Comparison of anticholinergic prescription rates (antipsychotic monotherapy vs. antipsychotic polypharmacy, SGA monotherapy vs. FGA monotherapy, and antipsychotic normal-dose vs. antipsychotic high-dose). #*p* < 0.01. FGA, first generation antipsychotic; SGA, second generation antipsychotic.

**FIGURE 3:**
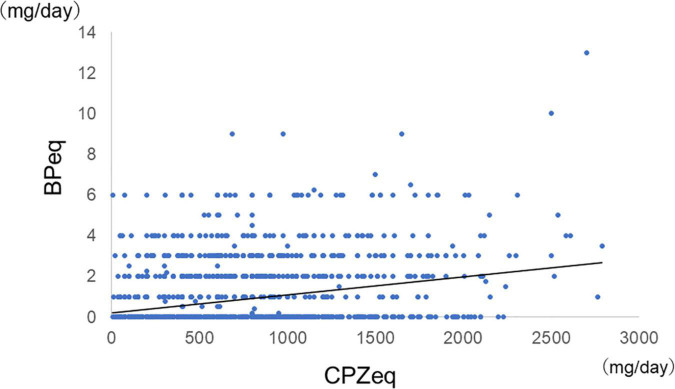
Correlation between antipsychotic dosage and dosage of anticholinergics (*n* = 2,027). rs = 0.33, *p* = 6.4 × 10^–15^. CPZeq, chlorpromazine equivalent; BPeq, biperiden equivalent.

**FIGURE 4:**
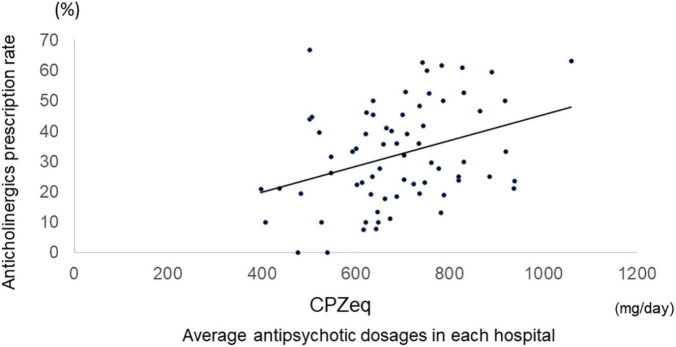
Correlation between antipsychotic average dosages in each hospital and anticholinergic prescription rate. rs = 0.34, *p* = 0.0004. CPZeq, chlorpromazine equivalent.

Among all samples, normal doses of antipsychotics had lower anticholinergic prescription rates than high doses (Antipsychotic high-dose: 43%, antipsychotic normal-dose: 27%, *p* = 2.0 × 10^–10^), and antipsychotic monotherapy had lower anticholinergic prescription rates than antipsychotic polypharmacy (antipsychotic monotherapy: 21%, antipsychotic polypharmacy: 43%, *p* = 2.2 × 10^–16^). Furthermore, among the monotherapy, FGA monotherapy had a higher anticholinergic prescription rate than SGA monotherapy (FGA monotherapy: 57%, SGA monotherapy 19%, *p* = 4.6 × 10^–15^) (Figure 2). A positive correlation was found between the antipsychotic dosage (CPZeq) and anticholinergics dosage (BPeq) [rs = 0.33, *p* = 6.4 × 10^–35^ (Figure 3)] and anticholinergic prescription rate in each hospital [rs = 0.34, *p* = 0.0004 (Figure 4)].

**FIGURE 5:**
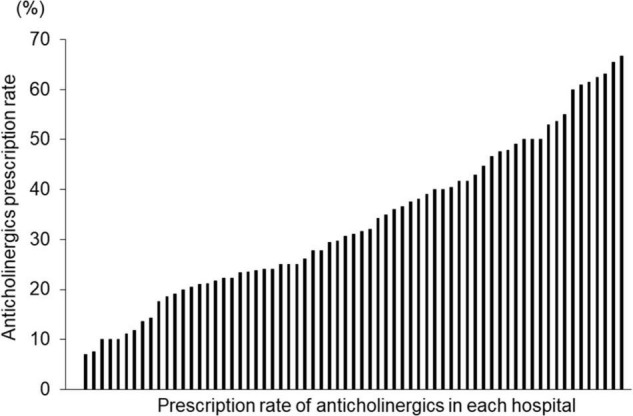
Proportion of patients with schizophrenia receiving anticholinergics in 69 hospitals. Vertical axis: Proportion of receiving anticholinergics in each hospital. Horizontal axis: Each hospital (*N* = 69).

There was a significant difference in the average proportion of patients receiving anticholinergics among the hospitals, ranging from 0 to 66.7% (Figure 5). The patients were classified into three groups according to the rate of anticholinergics prescriptions at each hospital. Among the three groups, there were significant differences in age, doses of antipsychotics, antipsychotics monotherapy rate, and prescriptions rate of anxiolytics/hypnotics and mood stabilizers; LG had significantly lower doses of antipsychotics and prescriptions rate of anxiolytics/hypnotics and significantly higher rates of antipsychotic monotherapy than MG and HG (Table 2).

**TABLE 2:** Differences in demographics between the three groups.

	LG (*n* = 415) mean (SD)	MG (*n* = 1,043) mean (SD)	HG (*n* = 569) mean (SD)	*P*	*Post-hoc*
Age (years)	45.3 (15.3)	46.5 (15.4)	44.5 (14.7)	0.032^a^	
Gender (% Female)	55.7%	53.3%	55.5%	0.59^b^	
CPZeq (mg/day)	599.9 (391.8)	689.5 (442.4)	733.5 (490.6)	1.9 × 10^–15a^	LG < MG = HG
Antipsychotic monotherapy (%)	70.8%	53.3%	49.9%	1.3 × 10^–11b^	LG < MG = HG
Benzodiazepines(%)	53.7%	71.2%	71.5%	1.8 × 10^–10b^	LG < MG < HG
Antidepressant (%)	7.5%	8.9%	8.4%	0.67^b^	
Mood stabilizer (%)	31.6%	37.6%	34.8%	0.087^b^	

*One-way analysis of variance (ANOVA) and Fischer’s exact test were used to make comparisons among the LG, MG, and HG with post-hoc Bonferroni.*

*CPZ-eq, chlorpromazine equivalent; LG, low rate group; MG, medium rate group; HG, high rate group.*

*^a^Reports F-value.*

*^b^Reports χ2-statistic.*

### Antipsychotic Dosage and Anticholinergic Prescription in Each Group

**FIGURE 6:**
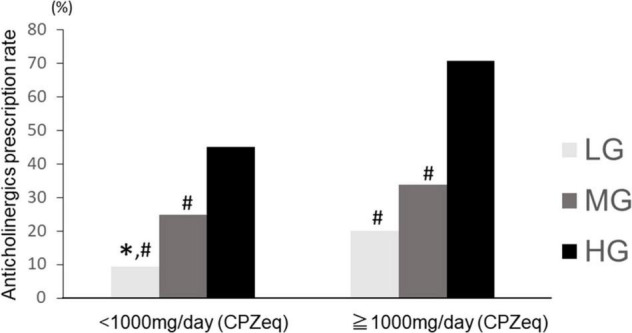
Anticholinergics prescription rate at high and normal doses of antipsychotics in the three groups. LG, low rate group; MG, medium rate group; HG, high rate group. Normal dose (CPZeq < 1,000 mg): LG (*n* = 350), MG (*n* = 816), HG (*n* = 439). High dose (CPZeq = 1,000 mg): LG (*n* = 65), MG (*n* = 227), HG (*n* = 130). ^∗^*p* < 0.033 vs. MG. #*p* < 0.033 vs. HG.

We divided the patients into two groups according to the antipsychotic dose: high-dose (≥1,000 mg/day CPZeq) and normal-dose (<1,000 mg CPZeq). The Chi-square test showed a significant difference (*p* < 0.01). The rate of anticholinergic prescriptions was significantly higher in the high-dose group for all LG, MG, and HG groups (LG: 9.4, 20.0%; MG: 24.8, 33.9%; and HG: 45.1, 70.8% for normal and high doses, respectively). When comparing the rate of anticholinergic prescriptions in these three groups, a significant difference (*p* = 2.2 × 10^–16^ and 1.4 × 10^–14^ for normal and high doses, respectively) was observed. In the normal-dose group, HG had a significantly higher anticholinergic prescription rate than LG and MG, and MG had a significantly higher anticholinergic prescription rate than LG. In the high-dose group, HG had a significantly higher anticholinergic prescription rate than LG and MG (Figure 6). Moreover, LG in the normal-dose group had a significantly lower anticholinergic prescription rate than MG (*p* = 8.5 × 10^–12^) and HG (*p* < 2.2 × 10^–16^) in the high-dose group and LG in the higher dose group had significantly lower anticholinergic prescription rate than HG (*p* = 0.003) in the normal-dose group. HG in the higher anticholinergic prescription rate than MG (*p* < 2.2 × 10^–16^) in the normal-dose group. HG in the normal dose group had significantly lower prescription rate in the HG in the higher-dose group (*p* = 2.2 × 10^–16^).

### Antipsychotic Prescription Pattern (Polypharmacy vs. Monotherapy) and Anticholinergic Prescription in Each Group

**FIGURE 7:**
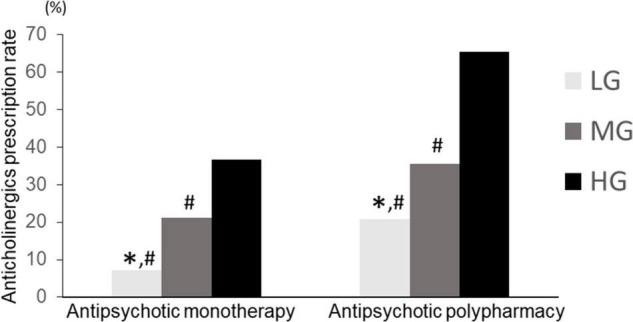
Anticholinergic prescription rate of LG, MG, and HG (antipsychotic monotherapy vs. antipsychotic polypharmacy). LG, low rate group. MG, medium rate group; HG, high rate group. Monotherapy, LG (*n* = 294), MG (*n* = 556), HG (*n* = 284). Polypharmacy: LG (*n* = 121), MG (*n* = 487), HG (*n* = 285). ^∗^*p* < 0.033 vs. MG. #*p* < 0.033 vs. HG.

We compared the anticholinergic prescription rates of the patients treated with antipsychotic monotherapy and polypharmacy. The Chi square test showed a significant difference (*p* < 0.01). In all previously considered groups, the rate of anticholinergics was significantly lower for patients with antipsychotic monotherapy than for those with antipsychotic polypharmacy (LG: 7.1, 20.7%, MG: 21.2, 35.5%, HG: 36.6, 65.3% for antipsychotic monotherapy and polypharmacy, respectively) (Figure 7). In the antipsychotic polypharmacy, LG had a significantly lower anticholinergics prescription rate than HG (*p* = 7.0 × 10^–15^), and HG had a significantly higher anticholinergic prescription rate than MG (*p* = 3.6 × 10^–14^). In the antipsychotic monotherapy group, LG had a significantly lower anticholinergics prescription rate than MG (*p* = 3.3 × 10^–6^) and HG (*p* = 2.7 × 10^–16^) (Figure 7). Moreover, LG in the antipsychotic polypharmacy had a significantly higher antipsychotic prescription rate than LG (*p* = 0.002) in the antipsychotic monotherapy. LG in the monotherapy had a significantly lower anticholinergic prescription rate than MG (*p* < 2.2 × 10^–16^) and HG (*p* < 2.2 × 10^–16^) in the antipsychotic polypharmacy. In this way, the characteristics of the proportion of anticholinergics prescriptions rate across the hospitals were maintained, regardless of the patients being on antipsychotic polypharmacy or monotherapy.

### Antipsychotic Monotherapy (First Generation Antipsychotic vs. Second Generation Antipsychotic) and Anticholinergic Prescription in Each Group

**FIGURE 8:**
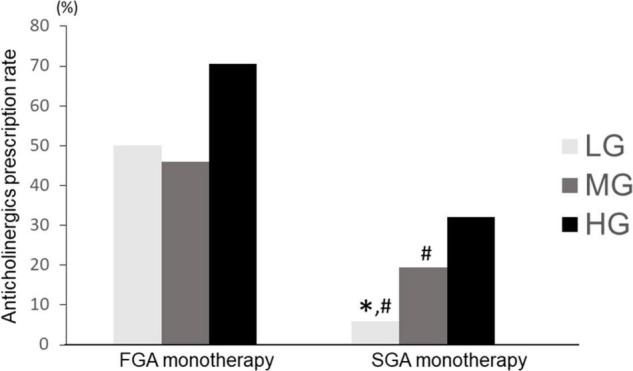
Comparison of anticholinergic prescription rates among LG, MG, and HG (SGA vs. FGA). LG, low rate group; MG, medium rate group; HG, high rate group; SGA, second generation antipsychotic; FGA, first generation antipsychotic. FGA monotherapy: LG (*n* = 8), MG (*n* = 37), HG (*n* = 34); SGA monotherapy: LG (*n* = 286), MG (*n* = 519), HG (*n* = 250); ^∗^*p* < 0.033 vs. MG. #*p* < 0.033 vs. HG.

Anticholinergics prescription rates were compared by dividing the antipsychotic monotherapy group into FGA monotherapy and SGA monotherapy. The Chi-square test showed a significant difference (*p* < 0.01). In the SGA monotherapy group, the anticholinergics prescriptions rate was significantly different among LG, MG, and HG groups (LG: 5.9%, MG: 19.5%, HG: 32.0%; *p* = 1.0 × 10^–14^). In a *post-hoc* analysis, LG and MG had significantly lower anticholinergic prescription rates than HG (*p* = 2.0 × 10^–7^, 5.4 × 10^–15^ for LG vs. HG and MG vs. HG, respectively) and MG had significantly lower anticholinergics prescription rates than HG (*p* = 5.6 × 10^–4^). Further, in the FGA monotherapy group, there was no significant difference in the rate of anticholinergics prescriptions among the three groups (LG: 50.0%, MG: 45.9%, HG: 70.6%; *p* = 0.104) (Figure 8). Moreover, LG in the SGA monotherapy had a significantly lower anticholinergic prescription rate than LG (*p* = 6.9 × 10^–4^), MG (*p* = 5.5 × 10^–6^), and HG (*p* = 2.0 × 10^–13^) in the FGA monotherapy. MG in the FGA monotherapy had a significantly higher anticholinergic prescription rate than LG (*p* = 1.1 × 10^–11^) and MG (*p* = 0.005) in the SGA monotherapy. HG in the SGA monotherapy had a significantly lower anticholinergic prescription rate than HG (*p* = 4.1 × 10^–14^) in the FGA monotherapy. Alternatively, treatment with SGA maintained the characteristics of the anticholinergics prescription rate among hospitals, whereas the FGA therapy did not follow any characteristics of anticholinergics prescription rate among hospitals.

### Anticholinergic Dosage in Each Group

A comparison of anticholinergic doses for each group in the categories described in “Antipsychotic Dosage and Anticholinergic Prescription in Each Group, Antipsychotic Prescription Pattern (Polypharmacy vs. Monotherapy) and Anticholinergic Prescription in Each Group, and Antipsychotic Monotherapy (First Generation Antipsychotic vs. Second Generation Antipsychotic) and Anticholinergic Prescription in Each Group” is presented in Supplementary Table 2. As with anticholinergic prescribing rates, dosages were higher for antipsychotics high dose, antipsychotic polypharmacy, and FGA use, as well as in hospitals that prescribed more anticholinergics (e.g., HG).

## Discussion

The results of this study show that anticholinergics were prescribed in approximately 30% of the considered patients with schizophrenia, which is similar to previous studies (35–38). In Japan, when anticholinergic drugs are used for schizophrenia patients, biperiden is often prescribed. To our knowledge, there is no clear evidence showing that biperiden is more effective or has fewer side effects than other anticholinergics. Biperiden was also the drug of choice in a previous study of the effects of anticholinergic drug reduction on cognitive function in Japanese patients with schizophrenia (39). Therefore, it appears that biperiden is often used in Japan for the treatment and prevention of extrapyramidal symptoms. In this study, we found that anticholinergic prescriptions varied by the hospital. Hospitals with high anticholinergic prescribing (e.g., HG) had high anticholinergic prescription rates for all prescribing patterns: dosage (high or normal), antipsychotic polypharmacy, and SGA monotherapy (Figures 6–8). In contrast, for FGA monotherapy, anticholinergic prescribing rates were high even in hospitals with fewer anticholinergic prescriptions (e.g., LG) (Figure 8). These results suggest that anticholinergic drugs may be prescribed even though they are not recommended in treatment guidelines, and the EGUIDE project is expected to optimize prescribing.

The purposes of the EGUIDE project are to educate psychiatrists about the treatment guidelines and to see if the quality of their clinical practice changes. In Asian studies (19, 37), the prescription rate for anticholinergic drugs is approximately 30%, similar to this study. Contrarily, studies in Europe have shown that the rate is <20% (40). This study showed that the prescription rate of anticholinergic drugs varied greatly from hospital to hospital. Particularly in hospitals classified as MG and HG groups, further education on anticholinergic prescription is needed. In addition, hospitals that were in the LG group will need to continue this status with education on the guidelines.

In general, antipsychotics have a dopamine D2 receptor blocking effect, and schizophrenia with EPS has a higher rate of D2 receptor blockade (41). A recent review reported that D2 receptor occupancy in the striatum greater than 78% increases the risk of EPS (42). In this study, we have also revealed a positive correlation between the antipsychotic dose and the anticholinergic use (Figure 3).

In this study, we divided the doses into high and normal doses and compared them across hospitals. The results of this study showed that hospitals with a higher frequency of anticholinergic usage had higher rates of anticholinergic prescription for both high and normal doses. Alternatively, rather than being the effect of antipsychotic dosage, the output may be more due to the prescribing habits of each hospital.

HG and MG also had a higher rate of antipsychotic polypharmacy. Consistent with the results of previous studies (43), the use of antipsychotic polypharmacy may be more likely to elicit EPS, resulting in increased use of anticholinergics. An interesting result of the present study is the observation that the anticholinergic prescribing rate is influenced by hospital characteristics, even if it is limited to antipsychotic monotherapy. This may be a consequence of the fact that hospitals that prescribe more anticholinergics (e.g., HG) either administer these prophylactically before the development of EPS or prescribe them without switching antipsychotics when EPS develops. In the current study, the monotherapy group was further divided into FGA monotherapy and SGA monotherapy for analysis, and in the SGA monotherapy group, anticholinergics were prescribed more frequently in hospitals with a higher prescribing rate, similar to the other findings of this study. Interestingly, there was no significant difference in anticholinergic prescription rates among the three groups in FGA monotherapy. This may be due to the pharmacological characteristics of FGA, i.e., when FGA is used as monotherapy, it must be prescribed even in hospitals that do not normally administer anticholinergics frequently (e.g., LG).

**FIGURE 9:**
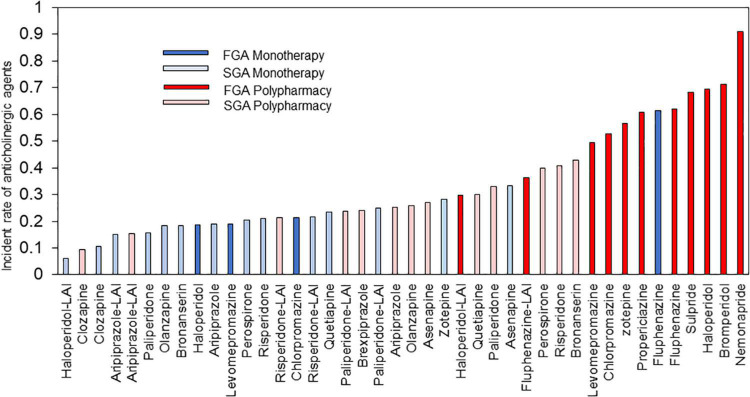
Anticholinergic drugs prescription rate of each antipsychotic. LAI, long-acting injection; FGA, first generation antipsychotic; SGA, second generation antipsychotic.

This study also revealed the proportion of concomitant anticholinergics use for each antipsychotic (Figure 9). Anticholinergics were used more frequently in FGA polypharmacy, followed by SGA polypharmacy, FGA monotherapy, and SGA monotherapy. Interestingly, there was the tendency of low rate of prescription of anticholinergics in patients with long-acting injection (LAI), which is in line with several previous studies suggesting that LAI has lower usage as antiparkinsonian drug than oral agents (44–46). Based on these results, it is advisable to plan prescriptions with SGA monotherapy. Regarding antipsychotics selection, it may be advisable to choose drugs that are less likely to produce extrapyramidal symptoms or to use LAI during the maintenance phase.

The current study presents some methodological strengths and limitations as follows. First, the absence of an evaluation of EPS rated by usual scales [e.g., Abnormal Inventory Movement Scale (AIMS) (47), UKU Side Effect Rating Scale (UKU) (48) or DIEPSS (49)] and the cross-sectional design did not allow us to evaluate the successful treatment of EPS with anticholinergics, and in how many patients, anticholinergics are further administered despite the absence of any EPS during long-term treatment, although the use of anticholinergics is connected to the presence of EPS in recent meta-analysis for antipsychotics (50). The EGUIDE project data also do not address side effects associated with anticholinergic drugs. Second, when evaluating the influence of antipsychotic treatment on anticholinergics users, we did not notice any specific effects of low-potency FGAs vs. high-potency FGAs, respectively, due to their different EPS liability. Third, other factors influencing anticholinergics prescription, such as the antipsychotic treatment duration, prescribing attitudes of the psychiatrists, and duration of anticholinergics use, were not surveyed. Fourth, the EGUIDE project focused on inpatients in several Japanese regions and sites; hence, the findings cannot apply to all patients, including outpatients, with schizophrenia in Japan. Further studies are required to overcome the drawbacks mentioned above. Despite the above limitations, the strengths of the study include all cases of patients with schizophrenia at discharge at a significant number of hospitals with >10 cases during the considered period; hence, the observed associations are unlikely to be due to chance.

Conclusively, institutional and biological (pharmacological) factors contribute to the prescription of anticholinergics. Institutional factors influence anticholinergic prescription in antipsychotic monotherapy, high dose of antipsychotics, and SGA monotherapy, whereas biological factors influence anticholinergics prescription in FGA monotherapy.

## Data Availability Statement

The datasets presented in this article are not readily available because, the data are not publicly available due to privacy and ethical restrictions (i.e., we did not obtain informed consent on the public availability of raw data). Requests to access the datasets should be directed to RH, ryotahashimoto55@ncnp.go.jp.

## Ethics Statement

The studies involving human participants were reviewed and approved by the ethics committees of the National Center of Neurology and Psychiatry and each participating university/hospital/clinic. The patients/participants provided their written informed consent to participate in this study.

## Author Contributions

HH, NY-F, and NHase were involved in data collection and data analysis and wrote the first draft of the manuscript. J-II, SO, KIc, RF, YK, YT, TT, FK, ToO, TsO, AM, and HKas were involved in the data analysis and contributed to the interpretation of the data and writing of the manuscript. HI, NHash, KOh, HisY, KOg, YY, HM, MU, SN, MT, HirY, TN, HT, MM, MK, EK, HKo, JM, CK, KM, and AH contributed to the interpretation of the data and data collection. KW and KIn were involved in the study design and contributed to the interpretation of the data. HKaw provided guidance and supported the research. RH supervised the entire project, collected the data, and was involved in the design, analysis, and interpretation of the data. All authors contributed to and approved the final article.

## Conflict of Interest

The authors declare that the research was conducted in the absence of any commercial or financial relationships that could be construed as a potential conflict of interest.

## Publisher’s Note

All claims expressed in this article are solely those of the authors and do not necessarily represent those of their affiliated organizations, or those of the publisher, the editors and the reviewers. Any product that may be evaluated in this article, or claim that may be made by its manufacturer, is not guaranteed or endorsed by the publisher.

## Supplementary Material

Supplementary Table 1 Differences in Top 3 anticholinergic prescribing rates in each group. LG, low rate group; MG, medium rate group; HG, high rate group; FGA, first generation antipsychotic; SGA, second generation antipsychotic.

Supplementary Table 2 Comparison of anticholinergic doses in each dosing pattern in the three groups. LG, low rate group; MG, medium rate group; HG, high rate group; FGA, first generation antipsychotic; SGA, second generation antipsychotic.

## Abbreviations

AIMS: abnormal inventory movement scale
BPeq: biperiden equivalent
CPZeq: chlorpromazine equivalent
DIEPSS: drug-induced extrapyramidal symptoms scale
EGUIDE: effectiveness of guidelines for dissemination and education in psychiatric treatment
EPS: extrapyramidal symptoms
FGA: first generation antipsychotic
LAI: long-acting injection
SGA: second generation antipsychotic
UKU: UKU side effect rating scale.
