# Supplementary material for: Prescription of Anticholinergic Drugs in Patients With Schizophrenia: Analysis of Antipsychotic Prescription Patterns and Hospital Characteristics
Source: Front Psychiatry. 2022 May 17;13:823826. doi: 10.3389/fpsyt.2022.823826 (PMC9152135; doi:10.3389/fpsyt.2022.823826)
Supplement: Supplementary Table 1 — Differences in Top 3 anticholinergic prescribing rates in each group. LG, low rate group; MG, medium rate group; HG, high rate group; FGA, first generation antipsychotic; SGA, second generation antipsychotic. [file Presentation_1.PPTX]

## Slide 1
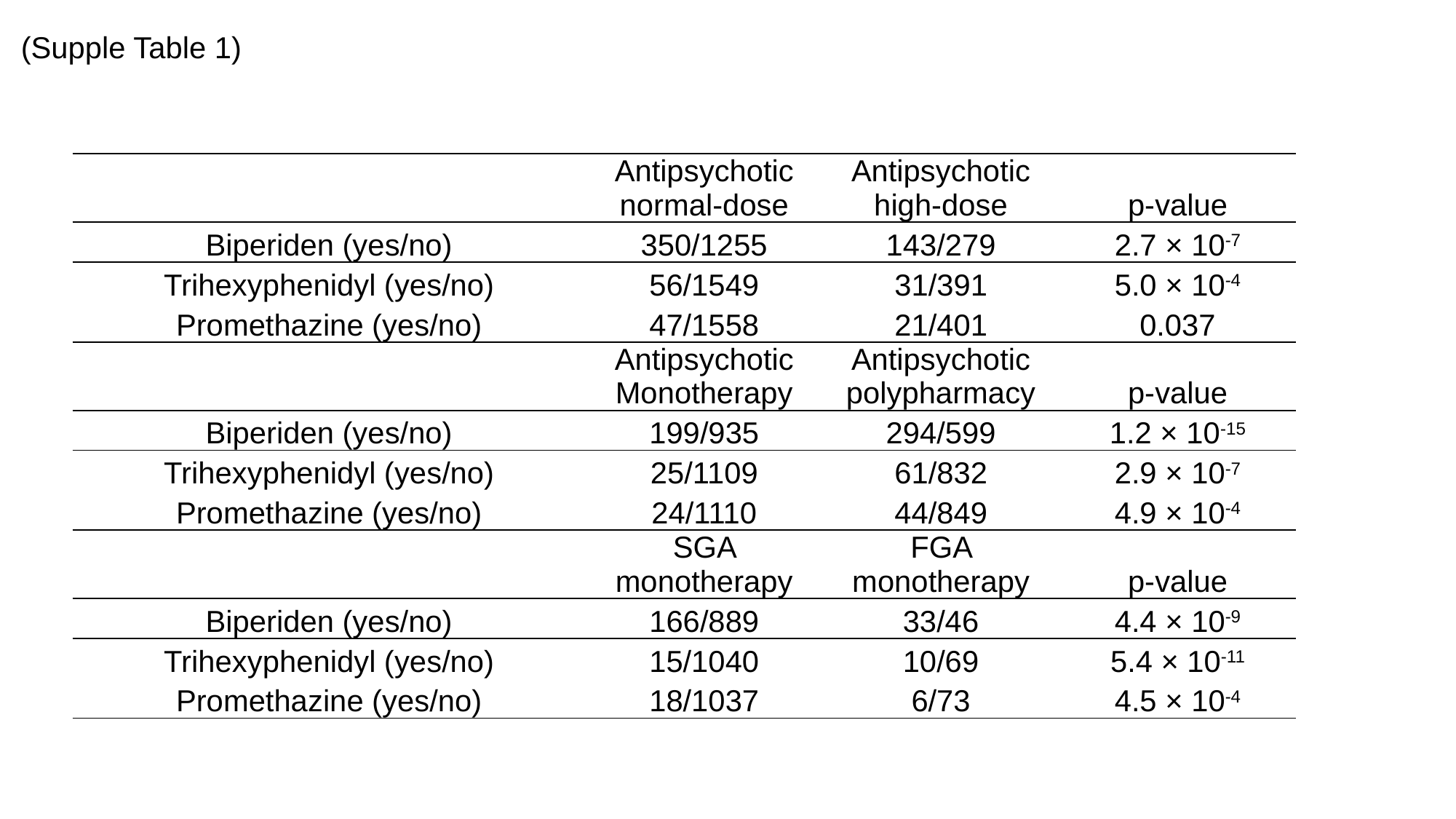

(Supple Table 1)
| | Antipsychotic normal-dose | Antipsychotic high-dose | p-value |
| --- | --- | --- | --- |
| Biperiden (yes/no) | 350/1255 | 143/279 | 2.7 × 10^-7^ |
| Trihexyphenidyl (yes/no) | 56/1549 | 31/391 | 5.0 × 10^-4^ |
| Promethazine (yes/no) | 47/1558 | 21/401 | 0.037 |
| | Antipsychotic Monotherapy | Antipsychotic polypharmacy | p-value |
| Biperiden (yes/no) | 199/935 | 294/599 | 1.2 × 10^-15^ |
| Trihexyphenidyl (yes/no) | 25/1109 | 61/832 | 2.9 × 10^-7^ |
| Promethazine (yes/no) | 24/1110 | 44/849 | 4.9 × 10^-4^ |
| | SGA monotherapy | FGA monotherapy | p-value |
| Biperiden (yes/no) | 166/889 | 33/46 | 4.4 × 10^-9^ |
| Trihexyphenidyl (yes/no) | 15/1040 | 10/69 | 5.4 × 10^-11^ |
| Promethazine (yes/no) | 18/1037 | 6/73 | 4.5 × 10^-4^ |

## Slide 2
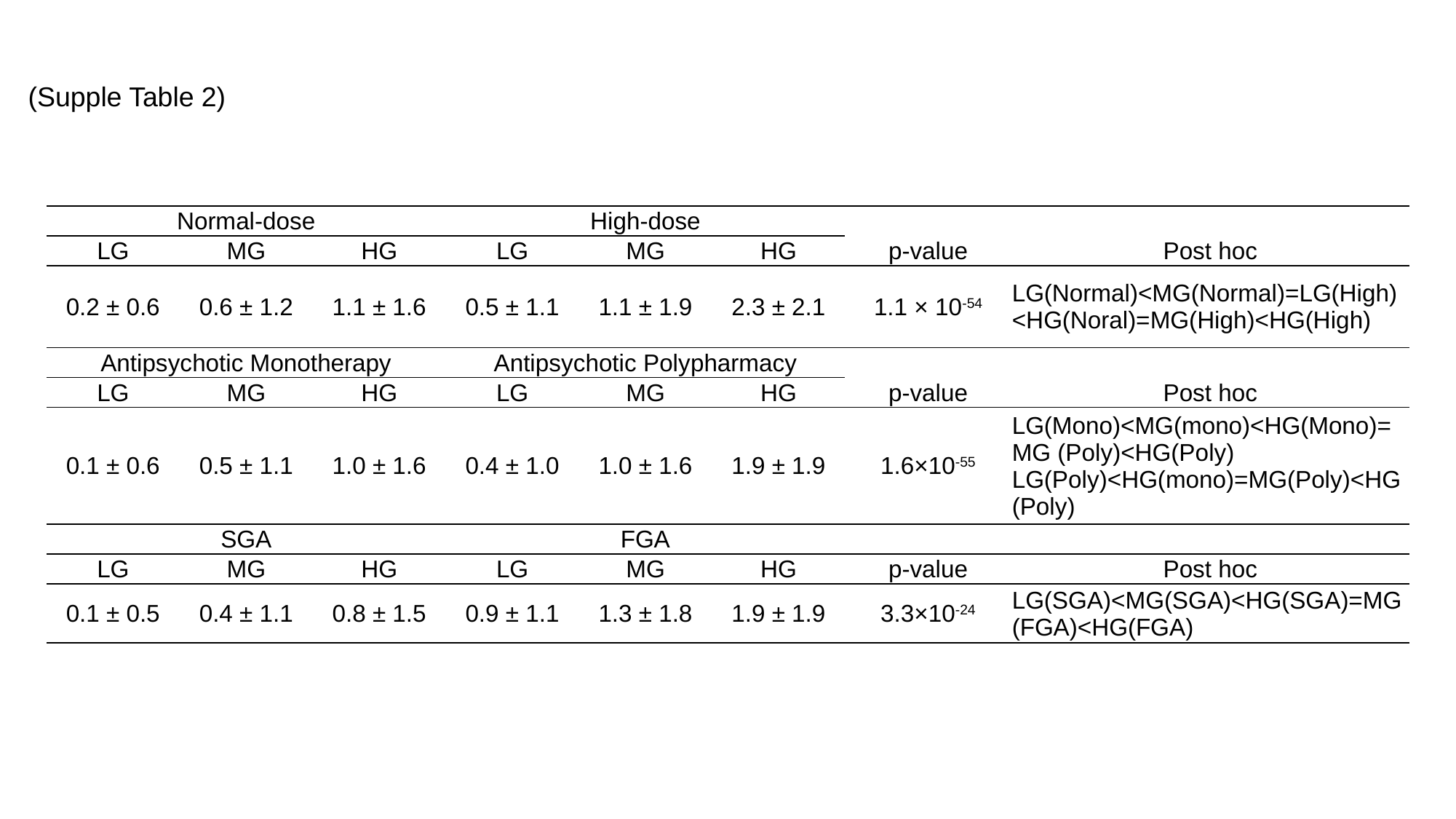

(Supple Table 2)
| Normal-dose | | | High-dose | | | | |
| --- | --- | --- | --- | --- | --- | --- | --- |
| LG | MG | HG | LG | MG | HG | p-value | Post hoc |
| 0.2 ± 0.6 | 0.6 ± 1.2 | 1.1 ± 1.6 | 0.5 ± 1.1 | 1.1 ± 1.9 | 2.3 ± 2.1 | 1.1 × 10^-54^ | LG(Normal)<MG(Normal)=LG(High)<HG(Noral)=MG(High)<HG(High) |
| Antipsychotic Monotherapy | | | Antipsychotic Polypharmacy | | | | |
| LG | MG | HG | LG | MG | HG | p-value | Post hoc |
| 0.1 ± 0.6 | 0.5 ± 1.1 | 1.0 ± 1.6 | 0.4 ± 1.0 | 1.0 ± 1.6 | 1.9 ± 1.9 | 1.6×10^-55^ | LG(Mono)<MG(mono)<HG(Mono)=MG (Poly)<HG(Poly) LG(Poly)<HG(mono)=MG(Poly)<HG(Poly) |
| SGA | | | FGA | | | | |
| LG | MG | HG | LG | MG | HG | p-value | Post hoc |
| 0.1 ± 0.5 | 0.4 ± 1.1 | 0.8 ± 1.5 | 0.9 ± 1.1 | 1.3 ± 1.8 | 1.9 ± 1.9 | 3.3×10^-24^ | LG(SGA)<MG(SGA)<HG(SGA)=MG(FGA)<HG(FGA) |
